# Genipin Enhances Kaposi’s Sarcoma-Associated Herpesvirus Genome Maintenance

**DOI:** 10.1371/journal.pone.0163693

**Published:** 2016-10-13

**Authors:** Miyeon Cho, Seok Won Jung, Soomin Lee, Kuwon Son, Gyu Hwan Park, Jong-Wha Jung, Yu Su Shin, Taegun Seo, Hyosun Cho, Hyojeung Kang

**Affiliations:** 1 College of Pharmacy and Cancer Research Institute and Institute of Microorganism, Kyungpook National University, Daegu, Republic of Korea; 2 Department of Medicinal Crop Research, National Institute of Horticultural and Herbal Science, Rural Development Administration, Eumseong, Republic of Korea; 3 Department of Life Science, Dongguk University, Goyang, Republic of Korea; 4 College of Pharmacy and Innovative Drug Center, Duksung Women's University, Seoul, Republic of Korea; Keck School of Medicine of the University of Southern California, UNITED STATES

## Abstract

Kaposi's sarcoma-associated herpesvirus (KSHV) is a Gammaherpesvirus that causes acute infection and establishes life-long latency. KSHV causes several human cancers, including Kaposi's sarcoma, an acquired immune deficiency syndrome (AIDS)-related form of non-Hodgkin lymphoma. Genipin, an aglycone derived from geniposide found in *Gardenia jasminoides*, is known to be an excellent natural cross-linker, strong apoptosis inducer, and antiviral agent. Although evidence suggests antiviral activity of genipin in several *in vitro* viral infection systems, no inhibitory effect of genipin on KSHV infection has been reported. Thus, our aim was to determine, using the iSLK-BAC16 KSHV infection system, whether genipin has inhibitory effects on KSHV infection. For this purpose, we evaluated biological effects of genipin on KSHV infection and finally determined the underlying mechanisms responsible for the bioactive effects of genipin. A cytotoxicity assay revealed that genipin caused 50% cytotoxicity at 49.5 μM in iSLK-puro (KSHV-negative) cells and at 72.5 μM in iSLK-BAC16 (KSHV-positive) cells. Caspase 3/7 activities were slightly suppressed by genipin treatment in iSLK-BAC16 cells while significantly induced in iSLK-puro cells. Production of the KSHV latency-associated nuclear antigen (LANA), but not that of the R-transactivator (RTA) protein, was significantly induced by genipin treatment at lower concentration. Consistent with the LANA upregulation, KSHV *LANA* transcripts, but not *RTA* transcripts, were expressed at a higher level. Furthermore, KSHV intracellular copy numbers were slightly increased at lower concentration of genipin, while KSHV extracellular copy numbers were significantly increased at higher concentration of genipin. Interestingly, genipin treatment at a lower concentration did induce the expression of DNA (cytosine-5)-methyltransferase 1 (DNMT1); however, a co-immunoprecipitation assay showed that the DNMT1 and LANA induced by genipin did not co-precipitate from iSLK-BAC16 cells. Moreover, a chromatin immunoprecipitation assay demonstrated that genipin treatment enhanced the binding of CCCTC-binding factor (CTCF) to the CTCF-binding site in the KSHV latency control region but suppressed the binding of structural maintenance of chromosomes protein 3 (SMC3) to this site. Genipin treatment also led to the recruitment of additional RNA polymerase to the majority of binding sites of some interesting proteins in the KSHV latency control region, which might be related to the extension of S phase in iSLK-BAC16 cells by genipin treatment. Finally, genipin treatment at lower concentration could promote the KSHV latent replication. In contrast, the treatment at higher concentration could induce the KSHV lytic replication. In conclusion, genipin was shown to be an interesting reagent, which we used to manipulate KSHV life cycle in KSHV latently infected cells.

## Introduction

Members of the *Herpesviridae* family are well-known viruses that can be found in many different species across the animal kingdom. Herpesviruses have a double-stranded DNA genome (124–230 kb) enclosed in an icosahedral capsid (~125 nm in diameter), which is composed of 162 capsomeres. Based on their biological properties, such as a host range, replication cycle, and cell tropism, these viruses are classified into the alpha, beta, and gamma herpesvirus subfamilies [1]. Kaposi's sarcoma-associated herpesvirus (KSHV, also known as HHV-8) is the eighth human herpesvirus, and it belongs to Gammaherpesviruses [2]. KSHV infection is associated with Kaposi's sarcoma (KS) and some B-cell malignancies such as an acquired immune deficiency syndrome (AIDS)-related form of non-Hodgkin lymphoma, called primary effusion lymphoma, and multicentric Castleman's disease [2]. Chemotherapy has been recommended for invasive KSHV-related diseases, and ganciclovir targeting KSHV replication has been used to inhibit KS development, despite the fact that the drug becomes useless once KS develops [3]. So far, the most effective therapy has been highly active antiretroviral therapy (HAART) that reduces HIV infection in AIDS–KS patients [4]. Although KSHV causes a wide range of human cancers, there are still not enough antiviral agents that specifically and effectively target KSHV.

Genipin, an aglycone derived from geniposide found in *Gardenia jasminoides*, is known to be an excellent natural cross-linker, strong apoptosis inducer, and effective antiviral agent [5]. For example, as a natural cross-linker, Hu et al. reported that genipin crosslinking of caseinophosphopeptide–chitosan nanoparticles significantly improved their stability and release profile in the gastrointestinal tract [6]. As a strong apoptosis inducer, genipin increased apoptosis in several types of cancer, including lung cancer, hepatocellular carcinoma, breast cancer, etc. [7]. The underlying mechanisms of apoptosis were found to be related to the activation of c-Jun N-terminal kinase (JNK) [8] and p38 mitogen-activated protein kinase [9], induction of reactive oxygen species [10], and signal transducer and activator of transcription 3 (STAT3) suppression [11]. As an effective antiviral agent, Lin et al. demonstrated that genipin could be an effective anti-influenza agent because of its enhanced binding affinity for the influenza M2 channel [12]. Simulations of M2-derivative complexes showed stable hydrogen bonding between genipin and M2 residues, Ser10 and Ala9. Tamura et al. have reported that genipin exhibits antiviral activity by inhibiting the nuclear export of the HIV Rev protein [13]. Recently, we have reported that genipin has potential as an antiviral agent by inducing Epstein–Barr virus (EBV) lytic reactivation in EBV-associated gastric carcinoma cells [14].

Antiviral agents can be categorized as virucides, antiviral chemotherapeutic agents, and immunomodulators [15]. Most antiviral agents affect actively replicating viruses but not their latent forms. In order to prevent KSHV from developing oncogenesis, it is necessary to identify a new antiviral drug that exerts inhibitory effects on viral latent replication because viral gene products produced during KSHV latent replication are likely to initiate viral oncogenesis. In this study, we tested if genipin could cause a significant biological effect on KSHV latent replication and evaluated if the compound could become a possible antiviral agent able to disturb KSHV latent infection. We found that genipin significantly intensified KSHV latent replication by upregulating the KSHV latency-associated nuclear antigen (LANA) and also intensified the recruitment of RNA polymerase II to the KSHV latency control region. These effects of genipin explain some of the mechanisms of action for this agent as a chemical modulator of the KSHV life cycle.

## Materials and Methods

### Preparation of genipin

Genipin (purity ≥ 98%) was obtained from Sigma–Aldrich (USA) and as a gift from Dr. Jeong from Duksung Women's University. Genipin was dissolved in dimethyl sulfoxide (DMSO) (Sigma–Aldrich), and a 200 mM stock solution was prepared. The solution was then filtered through a 0.22-μm filter (France) and stored at −20°C until use.

### Cell cultures

iSLK-BAC16 (endothelial cell line latently infected with KSHV, a gift from Jae J. Jung) was cultured in Dulbecco's Modified Eagle's Medium (Hyclone, Korea) supplemented with 10% fetal bovine serum (Hyclone), antibiotics/antimycotics (Gibco, USA), and GlutaMAX (Gibco) at 37°C, 5% CO_2_, and 95% humidity in a CO_2_ incubator [16]. iSLK-BAC16 cells were selected with hygromycin B (450 μg/mL) (Wako, Japan), G418 (120 μg/mL) (Sigma–Aldrich), and puromycin (1 μg/mL) (Sigma–Aldrich). iSLK-puro cells (a gift from Jae J. Jung) are KSHV-negative cells and were cultured in the same medium that was used for iSLK-BAC16 cells under selection of puromycin (5 μg/mL) [16]. The KSHV-positive PEL cell line BCBL1 was cultured at 37C° and 5% CO_2_ in RPMI medium (Gibco BRL) and supplemented with 10% fetal bovine serum and penicillin-streptomycin (50 U/mL).

### Cytotoxicity assay

Cytotoxic effects of genipin against iSLK-puro and iSLK-BAC16 cells were evaluated by cell cytotoxicity assays using Cell Counting Kit-8 (CCK-8) (Dojindo, Kumamoto, Japan), as described previously [17]. Briefly, 100 μL of a cell suspension (0.5 × 10^4^ cells) was seeded into each well of a 96-well plate. The cells were treated with a series of genipin concentrations on the following day and incubated for an additional 48 h. Then, 10 μL of CCK-8 solution was added to each well, followed by further incubation of the cells for 3 h, and absorbance was read at 450 nm using an ELISA reader. All steps of the procedure were conducted according to the manufacturer's recommended protocol. 50% cytotoxicity doses (CD_50_) of genipin against iSLK-puro and iSLK-BAC16 cells were determined.

### Viability assay

Quantitative analysis of cell count and viability was conducted using Muse^TM^ Count & Viability Kit as recommended by manufacturer. In briefly, iSLK-puro and iSLK-BAC16 cells were treated for 48 h with 49 μM genipin and 72 μM genipin, respectively. After genipin treatments, dead and floating cells were aspirated out. Resultant attached cells were washed twice with PBS before being trypsinized. The attached cells were then harvested by spinning down at 1200 rpm for 50 min. 0.5 x 10^5^ attached cells were subjected to the cell count & viability assay using the Muse^TM^ Cell Analyzer (Millipore, Darmstadt, Germany).

### Caspase 3/7 assay

To determine if genipin caused apoptosis, caspase 3/7 assays were conducted according to the protocol provided by the manufacturer (Promega, USA). iSLK-puro and iSLK-BAC16 cells were treated for 24 or 48 h with 50 and 100 μM genipin. Caspase 3/7 activities were measured in iSLK-BAC16 cells at 24 or 48 h using the Caspase Glo 3/7 kit (Promega, USA). Briefly, 100 μL of a 0.5 × 10^4^ cell suspension was seeded per well and treated with 50 and 100 μM genipin (range including CD_50_ concentration) on the following day. After 24 or 48 h of incubation, 100 μL of the caspase Glo 3/7 reagent was added. After 3 h of further incubation, luminescence of the cell suspension was measured at 560 nm.

### Annexin V & Dead Cell assay

To determine whether genipin induced apoptosis of iSLK-puro and iSLK-BAC16 cells, the Annexin V & Dead Cell Assay was conducted using Muse^TM^ Annexin V & Dead Cell Kit as recommended by manufacturer. In briefly, iSLK-puro and iSLK-BAC16 cells were treated with genipin at different concentrations for 48 h; iSLK-puro cells were treated with 0, 12, 24 and 49 μM genipin, while iSLK-BAC16 cells were treated with 0, 18, 36 and 72 μM genipin. After genipin treatments, dead and floating cells were aspirated out. Resultant attached cells were washed twice with PBS before being trypsinized. The attached cells were then harvested by spinning down at 1200 rpm for 50 min. 1.0 x 10^5^ attached cells were subjected to the MuseTM Annexin A & Dead Cell Kit on the Muse^TM^ Cell Analyzer. This application resulted in quantitative analyses of live, early and late apoptosis, and cell death on iSLK-puro and iSLK-BAC16 cells.

### Cell cycle analysis

Effects of genipin on the cell cycle progress of iSLK-puro and iSLK-BAC16 cells were assessed. iSLK-puro and iSLK-BAC16 cells were treated with 49.5 and 72.5 μM genipin (CD_50_ concentrations), respectively. Then, they were stained with propidium iodide (PI) solution after 48 h of treatment. Cell cycle analysis was performed using a fluorescence-activated cell sorter (FACS), Aria III (BD Bioscience, USA). Briefly, 1 × 10^6^ cells were seeded onto 6-cm culture dishes. On the following day, when the cells reached 70% confluence, they were treated with genipin and DMSO as a control. The cells were then washed with cold phosphate-buffered saline (PBS), fixed in 95% ethanol for at least 1 h, and then treated with 300 μg of RNase A to remove all traces of RNA. The cells were stained with 10× PI solution and analyzed for cell cycle progress using the Aria III FACS. Furthermore, to determine whether genipin affected cell cycle progress of iSLK-puro and iSLK-BAC16 cells, the cell cycle assay was conducted using the Muse^TM^ Cell Cycle Kit as recommended by manufacturer. In briefly, iSLK-puro cells and iSLK-BAC16 cells were treated with genipin at different concentrations for 48 h; iSLK-puro cells were treated with 0, 12, 24 and 49 μM genipin, while iSLK-BAC16 cells were treated with 0, 18, 36 and 72 μM genipin. After genipin treatments, dead and floating cells were aspirated out. Resultant attached cells were washed twice with PBS before being trypsinized. The attached cells were then harvested by spinning down at 1200 rpm for 50 min. 1.0 x 10^5^ attached cells were subjected to the Muse^TM^ Cell Cycle Kit on the Muse^TM^ Cell Analyzer for quantitative analysis of cell cycle progresses of iSLK-puro and iSLK-BAC16 cells.

### Real-time quantitative polymerase chain reaction

Total RNAs from iSLK-BAC16 cells treated with genipin at different concentrations were extracted using the RNeasy Mini Kit (Qiagen, Netherlands); iSLK-BAC16 cells were treated with 0, 18, 36 and 72 μM genipin. cDNA was then synthesized using Superscript II Reverse Transcriptase (Invitrogen, USA). Resultant cDNA was diluted 50-fold for the analysis of latent and lytic genes of KSHV. The effect of genipin on KHSV transcription was then evaluated by a real-time quantitative polymerase chain reaction (RT-qPCR) assay. The KSHV latent gene primers were specific for *K12* (*Kaposin*), *miRNA*, ORF71 (*vFLIP*), ORF72 (*vCyc*), and ORF73 (*LANA-1*), and the lytic gene primers were specific for ORF50 (*RTA*), ORF68, ORF69, *K14* (*vOX-2*), and ORF74 (*vGPCR*) (Table 1). The internal control gene primers were specific for the green fluorescent protein (*GFP*). Reverse transcription-quantitative PCR (RT-qPCR) was performed using the iQ SYBR Green reagent (Bio-Rad, USA). Each sample was analyzed in triplicate for the EBV gene expression. Expression data were normalized to the geometric mean of housekeeping gene *GAPDH* to control the variability in expression levels and were analyzed using the 2^-ΔΔCT^ method described by Livak and Schmittgen [18].

**Table 1:** Primer sets used in RT-qPCR to quantify the KSHV gene expression in iSLK-BAC16 cells.

KSHV Gene	Forward Primer Sequences(5’-3’)	Reverse Primer Sequences(3’-5’)
K12 (Kaposin)	ACAAACGAGTGGTGGTATCG	GTTTGTGGCAGTTCATGTCC
miRNA	ACGTGTTGCCAAGGTAGCATT	AGCCCGCTCATAAGACCATAAC
ORF71 (vFIP)	TGCGACCTGCACGAAACA	GGAGGAGGGCAGGTTAACGT
ORF72 (vCyc)	TGACGTTGGCAGGAACCA	CATTGCCCGCCTCTATTATCA
ORF73 (LANA-1)	GGATGGGATGGAGGGATTG	TTACCTCCACCGGCACTCTT
ORF50 (RTA)	CCTTCGGCCCGGAGTCT	CGGTTGCAGTTGCGTATACTCT
ORF68	GTGGTCGCATCCCACGA	ATGGACCCTGTGAGGTGTCTG
ORF69	TGCAGTGCAGGTACACACCA	GCATCTCGTCGGTGCAGTCT
K14 (vOX-2)	TGGTGGGCCTATTTGGGATA	GATGCACCGCCCTGCTT
ORF74 (vGPCR)	GCCCTCCTTATTCTGTTTTATGCT	CGCCTGGCTTGCAGCTT

### Western blot analysis

Effects of genipin on protein expression in iSLK-puro and iSLK-BAC16 cells were assessed using western blot analysis. Briefly, iSLK-puro and iSLK-BAC16 cells treated with genipin at different concentrations were harvested using trypsin at 48 h of treatment; iSLK-puro cells were treated with genipin at 0, 12, 24 and 49 μM, while iSLK-BAC16 cells were treated with 0, 18, 36 and 72 μM genipin. Cells (2 × 10^6^) were lysed using 100 μL of the reporter lysis buffer (Promega, USA) supplemented with 1 μL of the proteinase inhibitor (Sigma–Aldrich) and 10 μL of phenylmethylsulfonyl fluoride (PMSF) (Sigma–Aldrich). The cell lysates were further disrupted by sonication using a Bioruptor sonicator (Diagenode, Belgium) for 5 min, with 30-s on/off cycles. If necessary, the cell lysates were snap-frozen in liquid nitrogen and stored at −80°C. Cell lysates were resolved on a 10% sodium dodecyl sulfate polyacrylamide gel electrophoresis (SDS–PAGE) gel and subjected to western blot analysis. The following antibodies were used: anti-KSHV ORF50 (a gift from Dr. Izumiya at University of California, Davis, CA, USA), anti-KSHV ORF73 (Advanced Biotechnologies, Inc., USA), anti-GAPDH (Santa Cruz Biotechnology, Santa Cruz, CA, USA), anti-PARP (Cell Signaling Technology, USA), anti-BAX (Cell Signaling Technology), anti-Bcl-2 (Cell Signaling Technology), anti-cytochrome C (Cell Signaling Technology), anti-DNMT1 (Santa Cruz Biotechnology), anti-DNMT3a (Santa Cruz Biotechnology), anti-STAT3 (Cell Signaling Technology), and anti-phospho STAT3 (Cell Signaling Technology). Horseradish peroxidase (HRP)-conjugated goat anti-mouse IgG (Santa Cruz Biotechnology), HRP-conjugated goat anti-rabbit IgG (Santa Cruz Biotechnology), and HRP-conjugated goat anti-rat IgG (Bethyl Laboratories, USA) were used as secondary antibodies.

### Quantification of intracellular and extracellular KSHV genomic DNA copy numbers

To extract genomic DNA from iSLK-BAC16 cells treated with genipin at different concentrations, 1 x 10^6^ cells were seeded into a 6-cm plate, incubated for 48 h or 72 h, trypsinized, and collected as a cell pellet; iSLK-BAC16 cells were treated with 0, 18, 36 and 72 μM genipin. We then resuspended the cell pellets in 100 μL of FA lysis buffer [50 mM 4-(2-hydroxyethyl)-1-piperazineethanesulfonic acid (HEPES)–KOH, pH 7.5, 140 mM NaCl, 1 mM ethylenediaminetetraacetic acid (EDTA), pH 8, 1% Triton X-100, 0.1% sodium deoxycholate, 0.1% SDS, and protease inhibitors] and sonicated for 5 min, with 30-s on/off cycles. The protease inhibitors were added fresh every time (0.5 μL of proteinase inhibitor and 1 μL of PMSF per 100 μL of FA Lysis Buffer). The sonicated cells were mixed with 1 mL of RIPA lysis buffer (25 mM Tris–HCl, pH 7.6, 150 mM NaCl, 2% Nonidet P-40, 1% sodium deoxycholate, 10% SDS, and protease inhibitors) and treated with 400 μg of proteinase K for 2 h at 50°C, followed by treatment with 100 μg of RNase A for 30 min at 37°C. Proteins were extracted with one volume (approximately 500 μL) of phenol–chloroform–isoamyl alcohol (25:24:1) for 5 min at room temperature. The resultant supernatant was treated using the TaKaRa MiniBEST DNA Fragment Purification Kit to purify total genomic DNA. The resultant DNA (50 ng) was subjected to a quantitative PCR assay to access relative amounts of extracellular KSHV genome copy numbers. The extracellular amount of KSHV genomic DNA released by genipin treatment was determined relative to the amount of KSHV genomic DNA released by DMSO treatment (negative control) as an internal control. Each sample was analyzed in triplicate for measuring intracellular KSHV genome copy number. The genome copy numbers were normalized to the geometric mean of housekeeping gene *GAPDH* DNA fragment to control the variability in DNA amounts and were analyzed using the 2^-ΔΔCT^ method described by Livak and Schmittgen [18]. After that, the normalized genome copy numbers from the 18, 36, and 72 μM genipin treatments were compared with that from 0 μM genipin treatment. Relative extracellular KSHV copy numbers were measured using 20 mL of each culture medium collected from iSLK-BAC16 cells treated with genipin at different concentrations for 48 h or 72 h; iSLK-BAC16 cells were treated with 0, 18, 36 and 72 μM genipin. The culture media were filtered through a 0.45- μm syringe filter (Sartorius Stedim Biotech, France). The filtrates were loaded onto a 20% sucrose cushion in PBS and subjected to ultracentrifugation (CP100WX, Hitachi, Japan) at 27,000 rpm for 90 min. The viral pellet was lysed in 100 μL of FA lysis buffer and sonicated in the Bioruptor for 5 min with 30-s on/off cycles, followed by the genomic DNA extraction procedure described above. The extracted viral DNA was dissolved in 100 μL of RNase-free water. Each sample was analyzed in triplicate for measuring extracellular KSHV genome copy number. The genome copy numbers were analyzed using the 2^-ΔΔCT^ method described by Livak and Schmittgen [18]. After that, the analyzed genome copy numbers from the 18, 36, and 72 μM genipin treatments were compared with that from the 0 μM genipin treatment.

### KSHV infection assay

To determine whether genipin affected the KSHV infectivity against iSLK-puro cells, we infected iSLK-puro cells using KSHV virions derived from iSLK-BAC16 cells and counted the GFP foci on the infected cells. Briefly, we treated 10 × 10^6^ iSLK-BAC16 cells with either only sodium butyrate (1 mM) or both sodium butyrate (1 mM) and doxycycline (1 μg/mL) for 72 h. We then precipitated KSHV virions from the collected cell culture medium using a previously described method [19]. iSLK-puro cells (0.12 × 10^6^) were plated per well of 6-well plate as recipient cells for infection. In 24 h post incubation, they were treated with genipin, further incubated for 24 h, and then infected with KSHV in either low titer (less than 20 plaque-forming units of KSHV virions per well) or high titer (more than 100 plaque-forming units of KSHV virions per well). The cells were further incubated for 72 h, and finally the GFP foci formed on the iSLK-puro cells were counted. Each infection was analyzed four times in triplicate for calculating GFP foci formed through KSHV infection.

### Chromatin immunoprecipitation assay

Chromatin immunoprecipitation (ChIP) was performed according to the cross-linking chromatin immunoprecipitation (X-ChIP) protocol provided by Abcam (Cambridge, MA, USA), with a slight modification [20]. To determine interactions (recruitment patterns) of CCCTC-binding factor (CTCF), structural maintenance of chromosomes protein 3 (SMC3), RNA polymerase II (RNAP II), and phosphor-RNAP II (S5) with the LANA promoter, iSLK-BAC16 cells were treated with 72.5 μM genipin for 48 h. The Diagenode Bioruptor was used to sonicate genomic DNA into 200- and 400-base pair DNA fragments according to the manufacturer's protocol. The resultant cell lysates were subjected to immunoprecipitation with antibodies to CTCF (Millipore, Germany), SMC3 (Bethyl Laboratories), RNAP II (Abcam), phospho-RNAP II (S5) (Bethyl Laboratories), normal mouse IgG (Santa Cruz Biotechnology), normal rabbit IgG (Santa Cruz Biotechnology), and normal rat IgG (Santa Cruz Biotechnology). The precipitates were incubated with ChIP elution buffer (1% SDS, 100 mM NaHCO_3_); then the samples were decrosslinked at 65°C overnight and purified on Promega columns. The purified DNA was analyzed using RT-qPCR. ChIP values were calculated as fold increases over the isotype-specific IgG values for each antibody and primer set. The information on the primer sets used for this RT-qPCR analysis was reported previously [19] and will be provided upon request.

### Immunoprecipitation assay

Immunoprecipitation (IP) and western blot assays were performed according to the protocol described previously [20]. To analyze the interaction between LANA and DNA (cytosine-5)-methyltransferases (DNMTs), iSLK-BAC16 cells were treated with 72.5 μM genipin for 48 h. The cells were lysed in RIPA lysis buffer (25 mM Tris–HCl, pH 7.6, 150 mM NaCl, 2% Nonidet P-40, 1% sodium deoxycholate, 10% SDS, and protease inhibitors) on ice for 30 min, and the cell debris was cleared by centrifugation at 10,000 × *g* at 4°C. The resulting supernatants were immunoprecipitated with 2 μg of anti-KSHV ORF73 (Advanced Biotechnologies, Inc.), anti-DNMT1 (Santa Cruz Biotechnology), and anti-DNMT3a (Santa Cruz Biotechnology) antibodies at 4°C overnight. The immuno-complexes were collected with protein-A/G–Sepharose beads at 4°C for 2 h and washed with ice-cold lysis buffer three times. The immunoprecipitates were eluted with Laemmli sample buffer, and the samples were analyzed by a western blot assay.

### Statistical analysis

One-way ANOVA followed by Dunnet post test was applied to all groups of sample treatment for statistical analysis. In case of need, Two-tailed unpaired t test was also conducted in cases of need.

## Results

### Genipin induces cytotoxicity on iSLK-puro and iSLK-BAC16 cells

**Fig 1:**
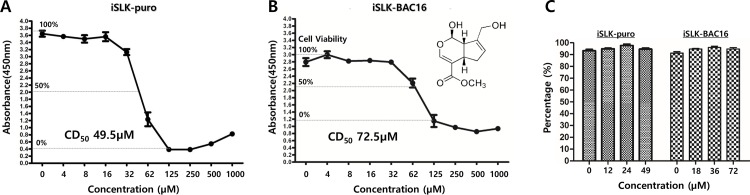
Effect of genipin on induction of cytotoxicity. A cytotoxicity assay was conducted to determine whether genipin produced cytotoxicity in iSLK-puro (KSHV-negative) and iSLK-BAC16 (KSHV-positive) cells. The iSLK-puro and iSLK-BAC16 cells were treated with a series of genipin concentrations for 48 h. The 50% cytotoxic doses (CD_50_ concentration) of genipin on iSLK-puro (A) and iSLK-BAC16 cells (B) were determined using the Cell Counting Kit (CCK-8; Dojindo, Japan). The percentages indicate the levels of survival of iSLK-puro and iSLK-BAC16 cells treated with a series of genipin concentrations. Upon determining CD_50_, the cell viability assay was conducted using the Muse^TM^ Count & Viability Kit (Millipore) to measure viability of attached cells that were separated from dead and floating cells (C).

We investigated whether genipin had cytotoxic effects on iSLK-BAC16 cells latently infected with KSHV. iSLK-puro cells were subjected to the cytotoxicity test as an internal control. The 50% cytotoxic doses (CD_50_) for genipin treatments were 49.5 and 72.5 μM against iSLK-puro and iSLK-BAC16 cells, respectively (Fig 1A and 1B). Next, in order to determine what percentage of cells did survive from the 49 μM genipin treatment for 48 h, the quantitative analysis of cell count and viability was conducted. When dead cells were removed from attached cells, more than 90% of attached cells were viable even after the 72 μM genipin treatment for 48 h (Fig 1C). These results suggested that the KSHV present in iSLK-BAC16 cells could be one of the main causes why iSLK-BAC16 cells were more resistant to genipin than iSLK-puro cells.

### Genipin did not significantly induce apoptosis in iSLK-BAC16 cells

**Fig 2:**
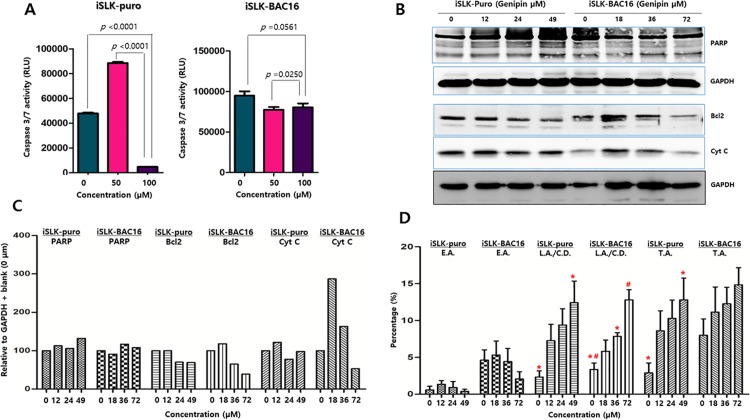
Effect of genipin on apoptosis. The effect of genipin on apoptosis was investigated by a caspase 3/7 assay, which indicates the apoptotic activity. (A) The caspase 3/7 assay demonstrated that the effects of genipin on apoptosis were significantly different in iSLK-puro and iSLK-BAC16 cells. Apoptosis was slightly suppressed in iSLK-BAC16 cells by the genipin treatment at both concentrations. (B) Western blot assays using antibodies to apoptosis signaling-related proteins, such as anti-PARP, anti-BAX, anti-Bcl-2, and anti-cytochrome C antibodies, were conducted to determine the effects of genipin on the induction of apoptosis in iSLK-puro and iSLK-BAC16 cells. (C) Intensities of bands in Western blot assay data were quantitated using GelQuant.NET program. Resultant band intensities standing for PARP, BAX, Bcl-2 and cytochrome C were normalized to the band intensity standing for GAPDH. After that, normalized intensities of bands from genipin treatments at different concentrations were compared with those from the 0 μM genipin treatments. (D) The cell cycle assay was conducted using the Muse^TM^ Cell Cycle Kit to determine whether the 48-h treatments of genipin at different concentrations made any effect on cell cycle progresses of iSLK-puro and iSLK-BAC16 cells. Results are averages of values from five independent experiments. Standard errors of the means are indicated with the T bars. *, # and ^ stand for statistical significant between two corresponding groups in ANOVA analysis.

The caspase-Glo 3/7 assay was conducted to test whether genipin had inhibitory or stimulatory effects on cell death of iSLK-puro and iSLK-BAC16 cells. Caspases are cysteine-aspartic and cysteine-dependent aspartate-directed proteases belonging to the family of cysteine proteases, which play essential roles in apoptosis (programmed cell death), necrosis, and inflammation [21]. iSLK-puro and iSLK-BAC16 cells were treated for 48 h with 50 or 100 μM genipin. Then they were followed by the measurement of caspase 3/7 activities. In iSLK-puro cells, the caspase 3/7 activities were significantly increased at the lower concentration but sharply decreased at the higher concentration (Fig 2A). However, in iSLK-BAC16 cells, the caspase 3/7 activities were slightly reduced at both concentrations (Fig 2A).

The western blot assay was conducted to determine the effects of genipin treatment on apoptosis signaling in iSLK-puro and iSLK-BAC16 cells. Since poly ADP ribose polymerase (PARP) is known to help cells maintain their viability [22], cleavage of PARP facilitates the cellular disassembly and serves as a marker for cells undergoing apoptosis. Compared to a mock (DMSO) treatment, the genipin treatments resulted in more cleavage of PARP proteins in iSLK-puro and iSLK-BAC16 cells (Fig 2B and 2C). In particular, the 49 μM genipin treatment to iSLK-puro cells produced clearly more cleaved PARP proteins than any other treatments tested. These results suggested that the genipin treatment at higher concentrations (49 or 72 μM) tends to induce more apoptosis in iSLK-puro cells than iSLK-BAC16 cells. The expression levels of the B-cell lymphoma 2 (Bcl-2) were affected by the genipin treatment. Bcl-2 (anti-apoptotic factor) were downregulated by genipin in iSLK-puro cells and iSLK-BAC16 cells (Fig 2B and 2C). However, the expression levels of Bcl-2-associated X protein (BAX) were also affected by the genipin treatment. BAX were regulated in an irregular manner by genipin in both cells (Fig 2B and 2C).

Furthermore, the Annexin V & Dead Cell Assay was conducted to determine whether genipin induced apoptosis in iSLK-puro and iSLK-BAC16 cells. Early apoptosis in both cells was not affected by genipin treatments at different concentrations (Fig 2D). Late apoptosis and cell death in both cells were significantly induced in a dose-dependent manner, suggesting that genipin induced late apoptosis and cell death regardless of KSHV infection (Fig 2D). However, genipin treatments at different concentrations induced total apoptosis in only iSLK-puro cells but not iSLK-BAC16 (Fig 2D). Taken together, these results suggested that the genipin treatment at higher concentration might induce cell death rather than apoptosis in iSLK-BAC16 cells.

### Genipin controls KSHV gene transcription

**Fig 3:**
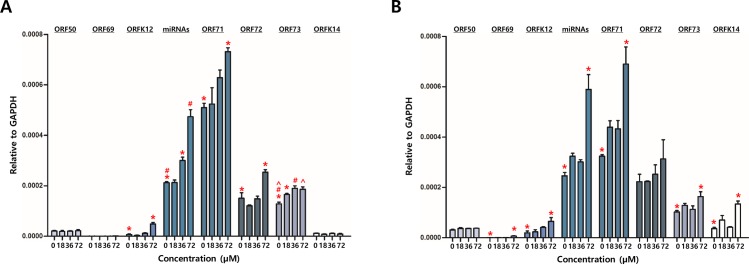
Effect of genipin on KSHV gene transcription. Effect of genipin on the KSHV gene transcription was determined by RT-qPCR assays. (A) Effect of genipin treatments at different concentrations for 48 h on the KSHV gene transcription. (B) Effect of genipin treatments at different concentrations for 72 h on the KSHV gene transcription. Results are averages of values from five independent experiments. Standard errors of the means are indicated with the T bars. *, # and ^ stand for statistical significant between two corresponding groups in ANOVA analysis.

To elucidate if genipin treatment affects the transcription of KSHV genes, transcription patterns of KSHV genes regulated by genipin treatment were determined by RT-qPCR assays. 48-h treatments of genipin showed to significantly enhance transcriptions of the KSHV ORF73, ORF72, ORF71, miRNA, and ORFK12 genes in a dose-dependent manner (Fig 3A). Those genes were most intensively transcribed when iSLK-BAC16 cells were treated for 48 h with 72 μM genipin. In similar, 72-h treatments of genipin also showed to significantly accelerate transcriptions of ORF69, ORFK12, miRNA, ORF71, ORF73, and ORFK14 genes in a dose-dependent manner (Fig 3B). Like the 48-h treatment, those genes were most intensively transcribed when iSLK-BAC16 cells were treated for 72 h with 72 μM genipin. These results suggested that the induction of KSHV latent gene expression was controlled by genipin in both a dose-dependent manner.

### Genipin controls the KSHV gene translation

**Fig 4:**
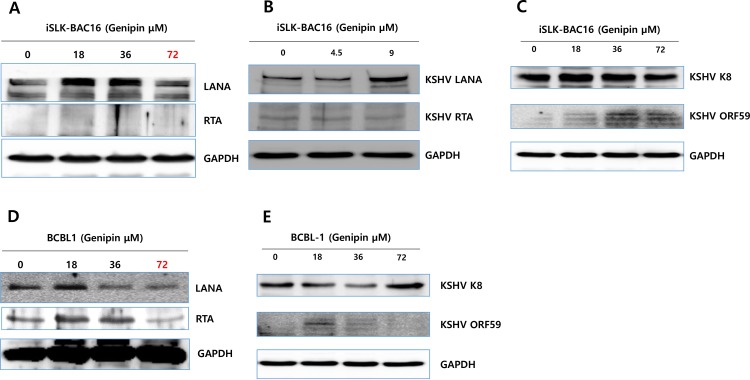
Effect of genipin on KSHV gene translation. Effects of genipin on KSHV gene translation were determined by Western blot assay. (A, B, C) Effect of genipin treatments at different concentrations for 48 h on productions of LANA, RTA, K8 and ORF59 in iSLK-BAC16 cells. (D, E) Effect of genipin treatments at different concentrations for 48 h on the productions of LANA, RTA, K8 and ORF59 in BCBL1 cells.

To elucidate if genipin treatment affects the translation of KSHV latent and lytic genes, Western blot assays were applied to evaluate the KSHV gene expression patterns in iSLK-BAC16 and BCBL1 cells regulated by genipin treatments. Genipin treatment to iSLK-BAC16 cells was shown to clearly induce the production of KSHV LANA encoded by KSHV ORF73 at 9 μM to 36 μM genipin treatment, while it did not induce the production of KSHV R-transactivator (RTA) encoded by KSHV ORF50 (Fig 4A and 4B). In fact, the KSHV LANA production in iSLK-BAC16 cells was greatest induced at 18 μM genipin treatment. However, the KSHV early lytic gene ORF59 in iSLK-BAC16 cells was upregulated at 36 μM to 72 μM genipin treatment, while other early lytic gene K8 was not induced by genipin treatments (Fig 4C). In similar, the 18 μM genipin treatment to BCBL1 cells showed to clearly induce production of LANA, RTA, and ORF59, while the 72 μM genipin treatment induced the KSHV K8 production in BCBL1 cells (Fig 4D and 4E). Taken together, these results suggested that genipin regulates the KSHV gene expression in a dose-independent manner.

### Genipin controls KSHV virion production

**Fig 5:**
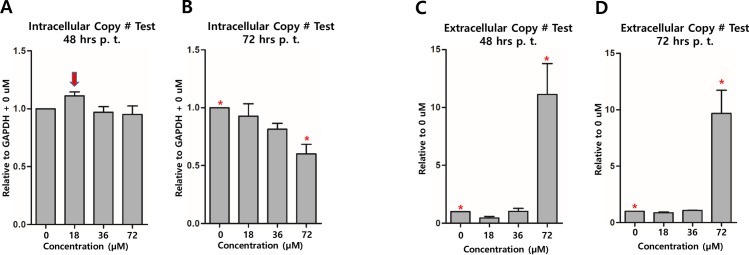
Effect of genipin on KSHV genome production. Effects of genipin on the production of intracellular and extracellular KSHV genomes were determined by KSHV genome copy number measurement assay. (A) Effect of genipin treatments at different concentrations for 48 h on the production of intracellular KSHV genomes in iSLK-BAC16 cells. (B) Effect of genipin treatments at different concentrations for 72 h on the production of intracellular KSHV genomes in iSLK-BAC16 cells. (C) Effect of genipin treatments at different concentrations for 48 h on the production of extracellular KSHV genomes in iSLK-BAC16 cells. (D) Effect of genipin treatments at different concentrations for 72 h on the production of extracellular KSHV genomes in iSLK-BAC16 cells. Results are averages of values from five independent experiments. Standard errors of the means are indicated with the T bars. *, # and ^ stand for statistical significant between two corresponding groups in ANOVA analysis.

To elucidate if genipin treatment affects the production of KSHV virion, KSHV genome copy numbers in iSLK-BAC16 cells treated with genipin at different concentrations were determined using qPCR assay. The 18 μM genipin treatment for 48 h induced the production of intracellular KSHV genomes even though it was not statistically significant (Fig 5A). In contrast, the 72 μM genipin treatment for 72 h significantly reduced the production of intracellular KSHV genomes (Fig 5B). Moreover, genipin treatments at lower concentrations (18 and 36 μM) for 48 h or 72 h did not affect the production of extracellular KSHV genomes (Fig 5C and 5D). However, the genipin treatment at higher concentration (72 μM) for 48 h or 72 h significantly increased extracellular KSHV genome copy numbers, meaning that the genipin treatment induced KSHV lytic reactivation from latent replication (Fig 5C and 5D). These results suggested that genipin treatments at lower concentrations are likely to maintain KSHV latent replication but the treatment at higher concentrations is likely to reactivate KSHV lytic replication.

### Genipin prevents KSHV infection

**Fig 6:**
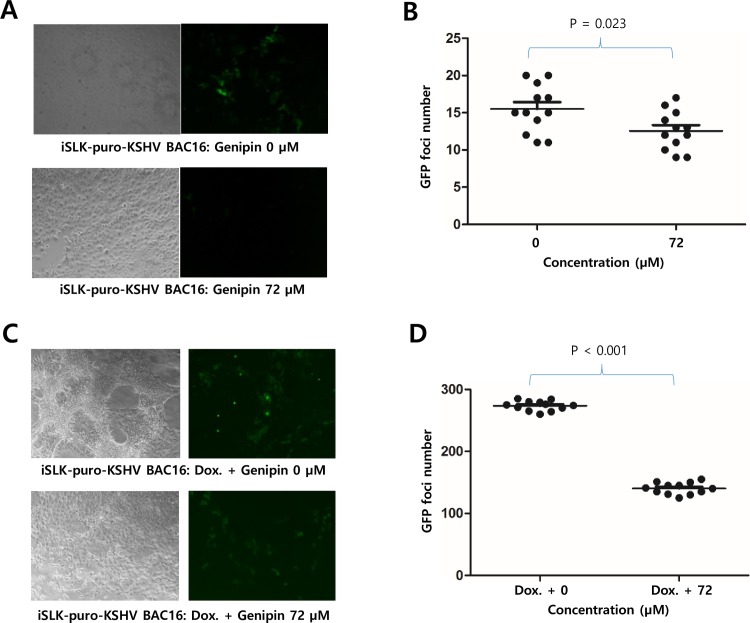
Effect of genipin on KSHV infection. Effects of genipin on KSHV infection to iSLK-puro cells were determined by KSHV infection assay. (A) GFP foci formed on 72 μM genipin treated iSLK-puro cells that were infected for 72 h with KSHV virions (low titer) harvested from iSLK-BAC16 cells treated with only sodium butyrate. (B) A graph showing the effect of genipin treatment at 72 μM on the low titer KSHV infection to iSLK-puro cells. (C) GFP foci formed on 72 μM genipin treated iSLK-puro cells that were infected for 72 h with KSHV virions (high titer) harvested from iSLK-BAC16 cells treated with both sodium butyrate and doxycyclin. (D) A graph showing the effect of genipin treatment at 72 μM on the high titer KSHV infection to iSLK-puro cells.

KSHV infection assay was conducted to elucidate if genipin treatment affects the prevention of KSHV infection. iSLK-BAC16 cells were in advance treated with only sodium butyrate (1 mM) for 72 h and resultant KSHV virions in media were harvested. At a day before infection, iSLK-puro cells were treated with 72 μM genipin and then infected for 72 h with KSHV virions. KSHV was in low titer because KSHV lytic reactivation was not fully induced by only sodium butyrate. GFP foci formed on iSLK-puro cells were evaluated under a fluorescent microscope. The 72 μM genipin treatment showed to decrease GFP foci numbers in iSLK-puro cells compared to the 0 μM treatment (Fig 6A and 6B). In addition, the iSLK-puro cells treated with 72 μM genipin were infected for 72 hours with KSHV virions harvested from iSLK-BAC16 cells treated with both sodium butyrate (1 mM) and doxycycline (1 μg/ml) as chemical KSHV lytic gene inducers. KSHV was in high titer because KSHV lytic reactivation was fully induced by both sodium butyrate and doxycycline. In similar, the 72 μM genipin treatment significantly decreased GFP foci numbers in iSLK-puro cells compared to the 0 μM treatment (Fig 6C and 6D). These results suggested that genipin treatment at higher concentration prevents KSHV infection.

### Genipin upregulates DNMT1 in iSLK-BAC16 cells

**Fig 7:**
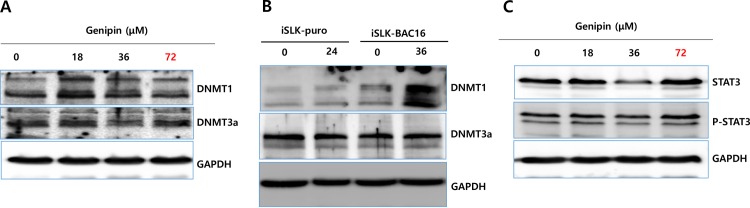
Effect of genipin on DNMT1. Effects of genipin on DNMT1 and DNMT3a were determined by western blot assays. (A) The western blot assays with anti-DNMT1 and anti-DNMT3a antibodies showing that lower concentrations of genipin, such as 18.12 and 36.25 μM, significantly induced DNMT1 but not DNMT3a production. (B) Specific upregulation of DNMT1 in iSLK-BAC16 cells that was determined by the Western blot assay using KSHV-negative iSLK-puro cells as a negative control. (C) Wstern blot assays with anti-STAT3 and anti-phospho-STAT3 antibodies showing that only a higher concentration of genipin, such as 72.5 μM, induced the STAT3 and phospho-STAT3 production in iSLK-BAC16 cells.

In order to evaluate if there were any epigenetic effects caused by genipin treatment, we conducted a western blot assay using anti-DNMT1 and anti-DNMT3a antibodies. When iSLK-BAC16 cells were treated with genipin at several concentrations, the DNMT1 production was highly enhanced in iSLK-BAC16 cells at lower concentrations of genipin, while the DNMT3a production was not affected (Fig 7A). Interestingly, the DNMT1 upregulation by genipin treatment was observed only in iSLK-BAC16 cells but not in iSLK-puro cells (Fig 7B). These results implied that DNMT1 upregulation by genipin treatment could be associated with KSHV infection. Further, since the STAT3 pathway is known to upregulate *DNMT1* [23], the DNMT1 upregulation by genipin suggested that the genipin treatment might stimulate the STAT3 signaling pathway. To examine this, western blot analysis using anti-STAT3 and anti-phospho-STAT3 antibodies was conducted. In our study, we could not observe that genipin treatment significantly induced the STAT3 and phospho-STAT3 expression, even though it strongly induced DNMT1 in iSLK-BAC16 cells. These results implied that the DNMT1 upregulation by genipin could be induced independently of the STAT3 signaling pathway (Fig 7C).

### Genipin weakens the protein–protein interactions between LANA and DNMTs

**Fig 8:**
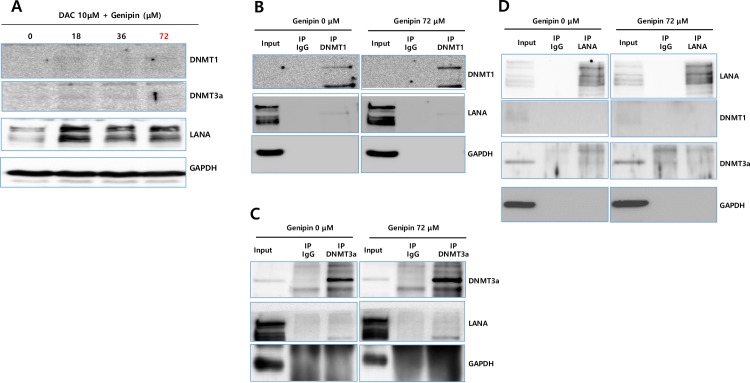
Effect of genipin on DNMT1 and LANA protein–protein interaction. Effects of genipin on the DNMT1 and LANA protein–protein interactions were determined by a DAC inhibitor assay and immunoprecipitation (IP)/western blot assays. (A) For the DAC inhibitor assay, the iSLK-BAC16 cells were first treated with DAC (5-aza-2'-deoxycytidine), a DNMT1 inhibitor, for 48 h, followed by the genipin treatment at different concentrations for 48 h. Then, a western blot assay was conducted, and it showed that under the inhibition of DNMT1 upregulation, the genipin treatment still upregulated LANA in the iSLK-BAC16 cells. (B) For the IP and western blot assays, the iSLK-BAC16 cells were first treated with genipin at 72.5 μM for 48 h. Then, proteins were immunoprecipitated with an anti-DNMT1 antibody, and DNMT1 and LANA were probed by western blot assays with anti-DNMT1 and anti-LANA antibodies. (C) The iSLK-BAC16 cells were treated with genipin at 72.5 μM for 48 h. Then, proteins were immunoprecipitated with an anti-DNMT3a antibody, and DNMT3a and LANA were probed by western blot assays with anti-DNMT3a and anti-LANA antibodies. (D) The iSLK-BAC16 cells were treated with genipin at 72.5 μM for 48 h. Then, proteins were immunoprecipitated with an anti-LANA antibody, and LANA, DNMT1, and DNMT3a were probed by western blot assays with anti-LANA, anti-DNMT3a, and anti-DNMT1 antibodies.

Since genipin treatment resulted in the upregulation of both KSHV LANA and DNMT1, it was important to determine whether the DNMT1 upregulation by genipin was correlated with the LANA upregulation. To examine this, we treated iSLK-BAC16 cells with genipin and 10 μM 5-aza-2'-deoxycytidine (DAC), a histone deacetylase inhibitor, for 48 h and then conducted a western blot assay to observe if there were any correlations between the DNMT1 and LANA upregulation. This assay showed that the DAC treatment could abolish the DNMT1 upregulation by genipin but could not abolish the LANA upregulation, suggesting that there was no regulatory correlation between the upregulation of the two proteins (Fig 8A). Moreover, since genipin upregulated LANA and DNMT1, it was questioned if genipin would have any effect on protein–protein interactions between LANA and DNTMs. To clarify this, we examined the possibility that genipin induced the LANA–DNMT interaction. Co-IP of DNMT1 and LANA could easily be demonstrated in an iSLK-BAC16 cell extracts (Fig 8B). The genipin treatment was shown to slightly weaken the protein–protein interaction between DNMT1 and LANA. When intensities of the bands standing for protein-protein interaction were quantified, the 72 μM geneipin treatment showed to enhance the DNMT1 expression by 134% compared to the 0 μM genipin treatment (100%), while the 72 μM treatment showed to decrease the interaction between DNMT1 and LANA by 62% compared to the 0 μM treatment (100%). On the other hands, co-IP of DNMT3a and LANA could not be easily demonstrated in iSLK-BAC16 cell extracts (Fig 8C). Next, reverse co-IP between LANA and DNMT1/DNMT3a was also conducted (Fig 8D). We could not observe any strong protein–protein interaction between LANA and DNMT1 but observed a weak protein–protein interaction between LANA and DNMT3a. The genipin treatment appeared to slightly weaken the LANA–DNMT3a interaction. These IP experiments suggested that the genipin treatment had a mild inhibitory effect on the LANA and DNMT protein–protein interactions.

### Genipin affects RNA polymerase recruitment to the LANA constant promoter

**Fig 9:**
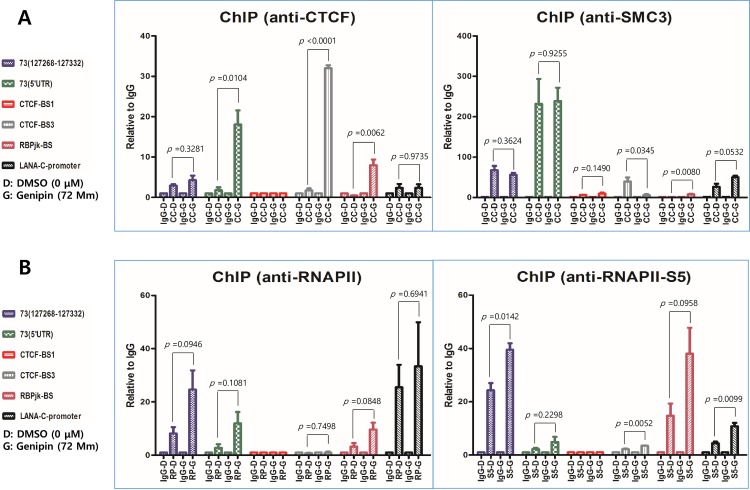
Effect of genipin on recruitment of RNA polymerase to the KSHV latency control region. Effects of genipin on the recruitment of RNA polymerase to the KSHV latency control region were determined by chromatin immunoprecipitation–qPCR assay. (A) The iSLK-BAC16 cells were treated with genipin at 72.5 μM for 48 h, then chromatin was immunoprecipitated with anti-CTCF and anti-SMC3 antibodies, and CTCF- and SMC3-binding DNA fragments were quantified by a qPCR assay. (B) Similarly, the iSLK-BAC16 cells were treated with genipin at 72.5 μM for 48 h, then chromatin was immunoprecipitated with anti-RNA polymerase II (RNAPII) and anti-S5 type RNA polymerase II (RNAPII-S5) antibodies, and the RNAPII- and RNAPII-S5-binding DNA fragments were quantified by a qPCR assay.

The RNA analysis suggested that genipin might affect the RNA polymerase function in transcription initiation or RNA processing of KSHV latency transcripts. To investigate this possibility, we performed ChIP assays to examine the effect of genipin on binding of several candidate proteins to the KSHV latency control region. We first examined the effect of genipin on binding of CTCF and the cohesion subunit SMC3 (Fig 9A). The genipin treatment caused significant increases in CTCF binding to the 5' untranslated region (UTR) of ORF73, CTCF-binding site 3, and RBP-Jk-binding site [19]. In contrast, the genipin treatment caused a clear decrease in SMC3 binding to CTCF-binding site 3. These results suggested that genipin treatment could affect the typical interactions between CTCF and cohesins, which might lead to the loss of transcriptional regulation of the CTCF–cohesin complex, as reported previously [19]. Further, to determine whether the levels of RNAP II were elevated at binding sites of several candidate proteins in the KSHV latency control region, we performed ChIP with antibodies specific for RNAP II and the S5 phospho-isoform of the C-terminal domain, using iSLK-BAC16 cells treated with genipin (Fig 9B). We found that the genipin treatment made RNAP II and its phospho-S5 form associated with polymerase pausing, and they were highly elevated at the ORF73, 5' UTR of ORF73, RBP-Jk-binding site, and LANA constant promoter. These findings suggested that the genipin treatment enhanced the recruitment of RNAP II at the KSHV latency control region to upregulate KSHV major latency genes.

### Genipin arrests the S to G2/M transition in the cell cycle of iSLK-BAC16 cells

**Fig 10:**
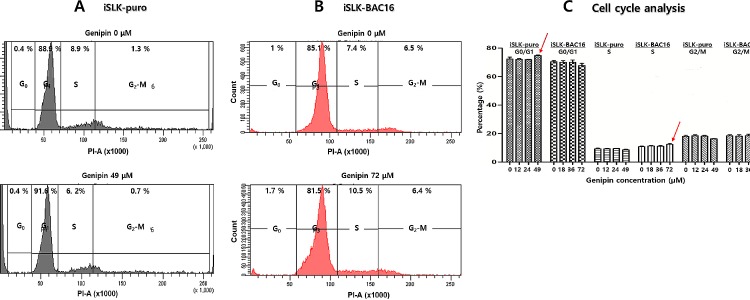
Effect of genipin on cell cycle progress. Effects of genipin on the cell cycle progress were determined using propidium iodide (PI) staining and FACS analysis. (A) The iSLK-puro cells were treated with either DMSO or genipin at 49.5 μM for 48 h, stained with PI, and analyzed by FACS. (B) The iSLK-BAC16 cells were treated with either DMSO or genipin at 72.5 μM for 48 h, stained with PI, and analyzed by FACS. (C) iSLK-puro cells and iSLK-BAC16 cells were treated with genipin at different concentrations for 48 h and then applied to Muse^TM^ Cell Cycle Kit on the Muse^TM^ Cell Analyzer for quantitative analysis of cell cycle progresses. Arrow bars point to both the G0/G1 stage of cell cycle of iSLK-puro cells treated with geneipin at 49 μM and the S stage of cell cycle of iSLK-BAC16 cells treated with geneipin at 72 μM.

Finally, since genipin significantly increased the KSHV intracellular copy numbers in iSLK-BAC16 cells, this implied that genipin might have biological effects on the regulation of S phase of the cell cycle. To examine this, we analyzed the biological effect of genipin on the cell cycle using PI staining and FACS analysis. These assays showed that the genipin treatment caused an arrest of the G1 to S transition in iSLK-puro cells; the G1 phase was increased by 2.7%, and the S phase was decreased by 2.7% (Fig 10A). In contrast, the genipin treatment caused an arrest of the S to G2/M transition in iSLK-BAC16 cells; the S phase was extended by 3.1%, and the G2/M phase was shortened by 0.1% (Fig 10B). Furthermore, genipin treatments at different concentrations for 48 h resulted in similar effects on cell cycle progresses of iSLK-puro and iSLK-BAC16 cells (Fig 10C). The genipin treatments at higher concentrations (49 and 72 μM) extended G0/G1 phase in iSLK-puro cells and S phase in iSLK-BAC16 cells even though statically not significant. These findings suggested that the genipin treatment stimulates DNA synthesis by extending the S phase, which may accelerate the synthesis of KSHV genome copies in iSLK-BAC16 cells.

## Discussion

We evaluated the biological effects of genipin on KSHV infection and determined the underlying mechanisms involved in the bioactive effects of genipin. A cytotoxicity assay revealed that the genipin treatment resulted in a 50% level of cytotoxicity at 49.5 μM in iSLK-puro (KSHV-negative) and 72.5 μM in iSLK-BAC16 (KSHV-positive) cells. The caspase 3/7 activities were slightly suppressed by the genipin treatment in iSLK-BAC16 cells while significantly induced in iSLK-puro cells. The KSHV LANA protein, but not the RTA protein, was produced at a higher level with genipin treatment, although both latent and lytic KSHV gene transcripts were upregulated. Furthermore, KSHV intracellular copy numbers were in slice increased by genipin treatment at lower concentration, while KSHV extracellular copy numbers were significantly increased by genipin treatment at higher concentration. Interestingly, the genipin treatment did induce the DNMT1 expression, besides that of LANA, but a co-IP assay showed that the proteins did not co-precipitate in iSLK-BAC16 cell lysates. Moreover, the chromatin IP assay demonstrated that the genipin treatment enhanced CTCF binding to the CTCF-binding site in the KSHV latency control region but suppressed SMC3 binding to this site. In addition to CTCF and SMC3, the genipin treatment recruited more RNA polymerase to most binding sites of some interesting proteins in the KSHV latency control region, which could be related to the extension of S phase in iSLK-BAC16 cells by the genipin treatment. Finally, it was suggested that the genipin treatment at lower concentration might accelerate the KSHV latent replication but the treatment at higher concentration might accelerate the KSHV lytic replication.

Based on previous studies, a lower dose of genipin tended to induce the JNK/Nrf2/ARE signaling pathway and consequently protect cells from cell death [24]. In contrast, the treatment with a high dose of genipin was shown to induce apoptosis via the p53-independent Egr1/p21 signaling pathway [25]. Therefore, these two studies suggested that genipin plays dual functional roles in the treated cells. One role is to protect cells from cell death by the genipin treatments at lower concentrations such as 9 μM and 18 μM. But, the other role is to induce cell death by the genipin treatments at higher concentrations such as 36 μM and 72 μM. Similar to the previous studies, our study also showed that the genipin played two opposite roles in the treated cells in a dose-dependent manner. The genipin treatment at lower concentration for 48 h significantly induced KSHV LANA production and slightly increased KSHV intracellular genome copy numbers, suggesting the genipin role in support of KSHV lytic replication.

The genipin treatment induced early apoptosis in the KSHV-negative cell line, iSLK-puro, through both activating caspases 3/7, while the KSHV-positive cell line, iSLK-BAC16 was protected from apoptosis through suppressing the caspase 3/7 activities. In addition, the genipin treatment steadily decreased Bcl2 expression in iSLK-puro cells in a dose-dependent manner, but the treatment increased Bcl2 expression at lower concentration and then decreased at higher concentrations in iSLK-BAC16 cells. These results implied that iSLK-BAC16 was more resistant to apoptosis induced by the genipin treatment. KSHV is known to encode viral Bcl-2 that is 60% identical to cellular Bcl-2, which has been shown to regulate apoptosis by dimerizing with other members of the family [26]. Therefore, due to the presence of vBcl-2, iSLK-BAC16 cells may be more resistant to apoptosis induced by genipin treatment than iSLK-puro cells, which do not have vBcl-2.

Wu et al. reported that KSHV interleukin-6 (IL-6) enhanced the expression of DNMT1 in endothelial cells, increased the global genomic DNA methylation, and promoted cell proliferation and migration [27]. The study suggested that vIL-6 plays a role in KS tumorigenesis, partly by activating DNMT1 and inducing aberrant DNA methylation. Similarly, genipin upregulated DNMT1 and increased the KSHV intracellular copy numbers. Thus, this enabled us to postulate that genipin might promote the KSHV latent infection by activating DNMT1 and inducing aberrant DNA methylation.

Since KSHV ORF73 gene is expressed very early after *de novo* infection, interacts with transcriptional regulators and chromatin remodelers, and regulates the LANA and RTA promoters, Hu et al. suggested that LANA contributes to the establishment of latency through epigenetic control. The study showed that LANA binds to multiple active viral and cellular promoters and associates with the H3K4 methyltransferase–hSET1 complex [28]. Our study showed that the 48-h treatment of genipin at lower concentration induced the upregulation of KSHV ORF73 gene in iSLK-BAC16 cells. This might have enabled the induced resultant LANA to upregulate itself, which subsequently resulted in the increase of KSHV intracellular copy numbers.

Alka et al verified that apoptosis triggers KSHV lytic replication independent of RTA and showed caspase activity is required to trigger KSHV replication [29]. They showed that when apoptosis triggers KSHV replication, the kinetics of late lytic gene expression is accelerated by 12 to 24 h and that KSHV virion produced following apoptosis has reduced infectivity. This study suggested that beside to the concentional pathway requiring RTA, there is an alternative apoptosis-triggered pathway that does not required RTA, has faster replication kinetics, and produces virus with lower infectivity. In similar, when iSLK-BAC16 cells were treated with genipin at 72 μM for 48 h and 72 h, KSHV KSHV virion productions were significantly induced independent of RTA expression. We repeated the extracellular KSHV genome copy number test several times and observed a similar result in each experiment; the 72 μM genipin treatment for 48 h or 72 h induced KSHV lytic replication without upregulating KSHV ORF50 gene. We could not find why and how this lytic induction would occur by the genipin treatment in detail. However, based on the previous study, one of our speculations is that the KSHV lytic reactivation by the 72 μM genipin treatment may be attained by initiating the alternative apoptosis-triggered pathway via the induction of late apoptosis and cell death by the 72 μM genipin treatment. However, we need to further study to clarify the reason why extracellular DNA are increased by the 72 μM genipin treatment.

Li et al. reported that CTCF and Rad21, a cohesin complex component, are potent restriction factors for KSHV replication, with the cohesion knockdown leading to 100-fold increases in viral yields [30]. Chen et al. also reported that the depletion of the cohesion subunits Rad21, SMC1, and SMC3 resulted in the lytic cycle gene transcription and viral DNA replication. In contrast, depletion of CTCF failed to induce the lytic transcription or DNA replication [31]. Our study showed that the genipin treatment weakened the cohesion and enhanced the CTCF recruitment at the CTCF/cohesin-binding site in the KSHV latency control region. Based on the above studies, the decrease of cohesion by genipin treatment would contribute to the induction of the KSHV lytic cycle gene transcription and viral DNA replication. However, our study showed that the genipin treatment did not induce the transcription of KSHV lytic and some latent genes but enhanced the KSHV intracellular copy numbers. Due to the upregulation of KSHV genes, including KSHV *LANA* encoded by ORF73, it might be necessary to recruit RNAP II to the KSHV latency control region. Our study showed that the genipin treatment enhanced the recruitment of RNAP II at several binding sites for transcription factors in the KSHV major latency control region.

At the same time, it was quite interesting to observe the tremendous increase in CTCF at the KSHV major latency control region, induced by genipin treatment. Since there have been no previous reports showing any biological effect resulting from the induction of a CTCF increase, it was difficult to infer any effect of the increased CTCF on KSHV biology. However, one of our postulates was that the increased CTCF might not promote the lytic gene transcription induced by the depletion of cohesins. Instead, the increased CTCF might suppress the induction of KSHV lytic gene transcription by cohesin depletion and additionally contribute to maintaining the KSHV latent replication. Thus, given that the genipin treatment produced more KSHV LANA transcripts and protein, the increased CTCF level in our study might tremendously contribute to the suppression of the KSHV lytic reactivation and maintain the KSHV latent replication in the presence of genipin.

Fruits of *G*. *jasminoides* are most popular as a tea for the treatment of certain disorders. Genipin contained in large amounts in these fruits is known to affect a diverse range of bioactivities [7]. Our study demonstrates that genipin acts as a bioactive agent modulating the KSHV life cycle. Due to the possibility that the fruit of *G*. *jasminoides* might protect host cells from the KSHV latent infection, fruits of *G*. *jasminoides* can be candidates for medicinal foods used to prevent KSHV-associated disorders. This study highlights a fundamental possibility of the development of *G*. *jasminoides* as a medicinal food containing genipin as a major bioactive compound against KSHV infection and its related cancers.
