# Correction: Genipin Enhances Kaposi's Sarcoma-Associated Herpesvirus Genome Maintenance

**DOI:** 10.1371/journal.pone.0167185

**Published:** 2016-11-18

**Authors:** Miyeon Cho, Seok Won Jung, Soomin Lee, Kuwon Son, Gyu Hwan Park, Jong-Wha Jung, Yu Su Shin, Taegun Seo, Hyosun Cho, Hyojeung Kang

In the Funding section, the grant number from the funder “The Ministry of Education, Science and Technology” is listed incorrectly. The correct grant number is: Project No. 2016R1A6A1A03007648
